# Improved ferroelectric properties and band-gap tuning in BiFeO_3_ films *via* substitution of Mn

**DOI:** 10.1039/c9ra05914h

**Published:** 2019-09-17

**Authors:** Song Yang, Guobin Ma, Lei Xu, Chaoyong Deng, Xu Wang

**Affiliations:** Guizhou Key Laboratory for Characteristics of Electronic Functional Composites, College of Big Data and Information Engineering, Guizhou University Guiyang 550025 P. R. China SongYang_gzu@126.com

## Abstract

Multiferroic BiFe_1−*x*_Mn_*x*_O_3_ (*x* = 0, 0.04, 0.08, 0.12) films have been prepared on Pt/Ti/SiO_2_/Si and ITO/glass substrates *via* the solution-gelation technique. The impacts of Mn doping of BFO thin films on the structure, morphology, leakage current, ferroelectric properties and optical band gap have been systematic investigated. From the XRD patterns, all samples match well with the perovskite structure without an impurity phase and the thin films exhibit dense and smooth microstructure. A leakage current density of 1.10 × 10^−6^ A cm^−2^ which is about four orders of magnitude lower than that of pure BiFeO_3_ was observed for the 8% Mn doped BFO thin film at an external electric field <150 kV cm^−1^. An increase in the remnant polarization with Mn substitution was observed, with a maximum value of ∼19 μC cm^−2^ for the 8% Mn-substituted film. Moreover, optical absorption spectra indicate that the doping of Mn has an effect on the energy band structure. Compared with pure BiFeO_3_, Mn doped thin films present an intense red shift as shown in the UV-visible diffuse absorption together with the decreased direct and indirect optical band gaps. In addition, this work gives insight into the relationship between ferroelectric remnant polarization and band-gap and finds that the optical band gap decreases with the increase of residual polarization.

## Abstract 2

Multiferroic BiFe_1−*x*_Mn_*x*_O_3_ (*x* = 0, 0.04, 0.08, 0.12) films have been prepared on Pt/Ti/SiO_2_/Si and ITO/glass substrates *via* the solution-gelation technique.

## Graphical abstract

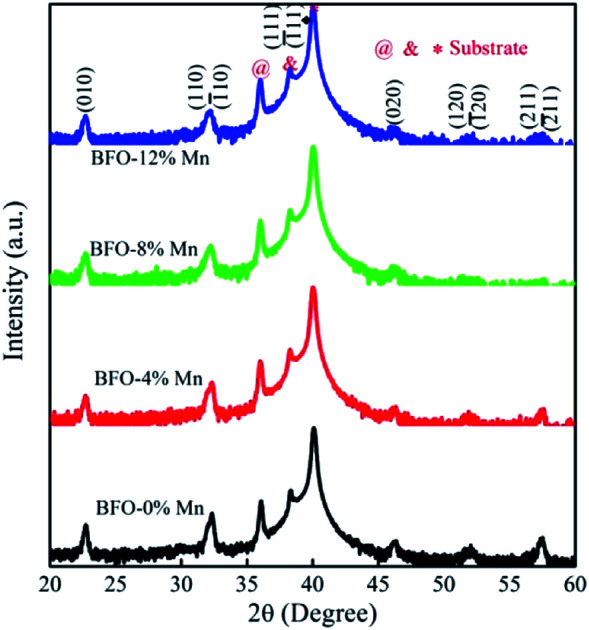

## 1. Introduction

Multiferroic materials, which possess simultaneous ferroelectric, ferromagnetic, and ferroelastic ordering, have attracted much attention. Among these materials, BiFeO_3_ (BFO) is the only single phase multiferroic material with both a high ferroelectric Curie temperature (*T*_C_ ∼ 1103 K) and a high antiferromagnetic Néel temperature (*T*_N_ ∼ 643 K).^1–3^ It is also a known Pb-free and environmentally friendly material.^4–6^ Additionally, BFO thin films exhibit remarkable ferroelectric photovoltaic effects.^7^ Compared to most classical ferroelectric materials, BFO has a small band gap with reported values in the range of 2.6–3.0 eV,^8^ and a very large remnant ferrroelectric polarization, which can offer exciting opportunities for use in both optoelectronics and solar energy devices towards abundant renewable clean energy harvesting.^9^ However, the application of BFO is seriously hindered due to its high leakage current.^4^ Some reports also showed weak polarization in BFO thin films, which is attributed to the presence of impurity phases and oxygen vacancies.^10^ To overcome these problems significant efforts have been made, the substitution technology at the A or B position of perovskite crystals is the most widely used.^11–13^ Meanwhile, further improve the photovoltaic characteristics, it is necessary to narrow band-gap of the ferroelectric materials with larger polarization and smaller leakage. Therefore, in this work, thin films BiFe_1−*x*_Mn_*x*_O_3_ (BFMO, *x* = 0, 0.04, 0.08, 0.12) were prepared on Pt/Ti/SiO_2_/Si and ITO/glass substrates by the solution-gelation technique. The microstructure, surface morphologies, ferroelectric and photovoltaic band-gap of BFO and Mn-doped BFO were discussed in detail.

## 2. Experiment

The BFMO thin films were prepared on Pt/Ti/SiO_2_/Si and ITO/glass substrates by the solution-gelation technique. The precursor solutions were fabricated by using bismuth nitrate [Bi(NO_3_)_3_·5H_2_O] (99%), iron nitrate [Fe(NO_3_)_3_·9H_2_O] (98.5%) and manganese acetate [Mn(CH_3_COO)_2_·4H_2_O] (99%) as starting materials and ethylene glycol methyl ether (99.5%) as solvent. Acetic anhydride was also added as dehydrating agent. 10 mol% of excess Bi was added to compensate for bismuth loss during the heat treatment. Then the solution was stirred at the right temperature for several hours to form a homogeneous sol. The resultant solutions with concentration of 0.3 mol L^−1^ were deposited on substrates by spin-coating with the velocity 4000 rpm for 30 s. The as-deposited wet thin films were pre-annealed at 450 °C for 180 s and crystallized at 600 °C for 300 s in a rapid thermal process furnace. The spin-coating and thermal treatments were repeated several times to obtain the desired film thickness.

The crystalline structures of the films were analyzed by X-ray diffraction (XRD, D/max-2500 V Rigaku) with Cu-Kα radiation. The surface morphologies were investigated by an atomic force microscope (AFM, Bruker MultiMode 8). The ferroelectric and leakage current properties were measured using a multiferroic tester system (MultiFerroic 200 V, Radiant Technologies). The optical absorption spectrum was measured by an ultraviolet-visible spectrophotometer (Hitachi U-4100).

## 3. Results and discussion

### 3.1. XRD analysis

**Fig. 1:**
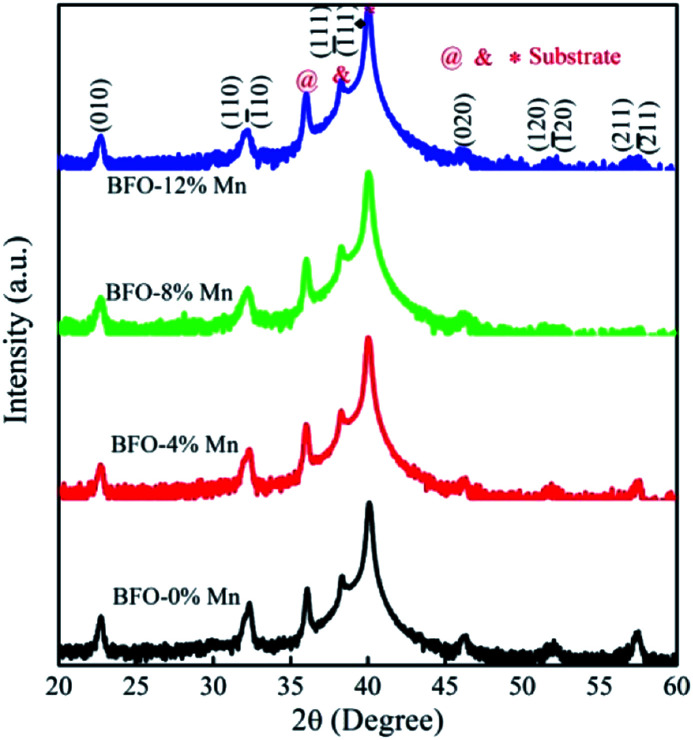
X-ray diffraction patterns of Mn substituted BFO thin films.

Fig. 1 shows the X-ray diffraction patterns (XRD) of Mn substituted BFO thin films grown on the Pt/Ti/SiO_2_/Si substrates. All diffraction peaks of the thin films exhibit a rhombohedral perovskite structure with (111) orientation, which is good agreement with the ICDD (PDF # 72-2112). No any other intermediate phases like Bi_2_Fe_4_O_9_ (ref. 14) has been found because the ionic radii of manganese are similar to that of Fe^3+^ (demonstrated in Table 1 (ref. 15)). It indicates that all these thin films are single-phase BiFeO_3_ materials in the same structure and the doping does not affect the lattice symmetry. In fact, their extranuclear structure of the outermost two layers exhibits high similarity.^16^ The (111) and (1̄11) peaks overlap completely and the intensity remains roughly the same, while the (120) and (1̄20) peaks also overlap completely and the intensity is decreasing gradually. Since that the dopants with different ionic radius could cause the distortion of crystal lattice to various degrees and even change their syngony.^17^ Futhermore, all films show the (111) preferred orientation for the (111)-Pt/Ti/SiO_2_/Si substrate.

**Table 1:** Radius of common ions quoted from Lange's Chemistry Handbook, Version 15 (ref. 15)

Ion	Bi^3+^	Fe^3+^	Mn^3+^	O^2−^
Radius/nm	0.096	0.055	0.058	0.132

The average crystallite size of the samples were calculated by using Scherer's formula:^18^1
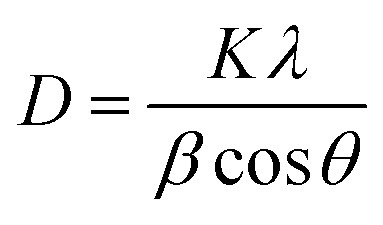
where *K* is the shape factor (0.89), *λ* is the wavelength of Cu (Kα), *β* is the full width half maxima (in radians), *θ* is the angle of diffraction, and the data is shown in Table 2. It was observed that crystallite size increase with Mn-doping from *x* = 0 to 0.08 in BiFe_1−*x*_Mn_*x*_O_3_.

**Table 2:** Lattice parameters calculated from the X-ray diffraction patterns and RMS roughness of Mn substitued BFO thin films

Mn concentration (*x*)	Average crystallite size (*D*) nm	RMS roughness (*R*_q_) nm
0	19.7	15.6
0.04	22.65	11.2
0.08	23.96	8.6
0.12	21.08	10.4

### 3.2. Surface morphology and domain structure analysis

**Fig. 2:**
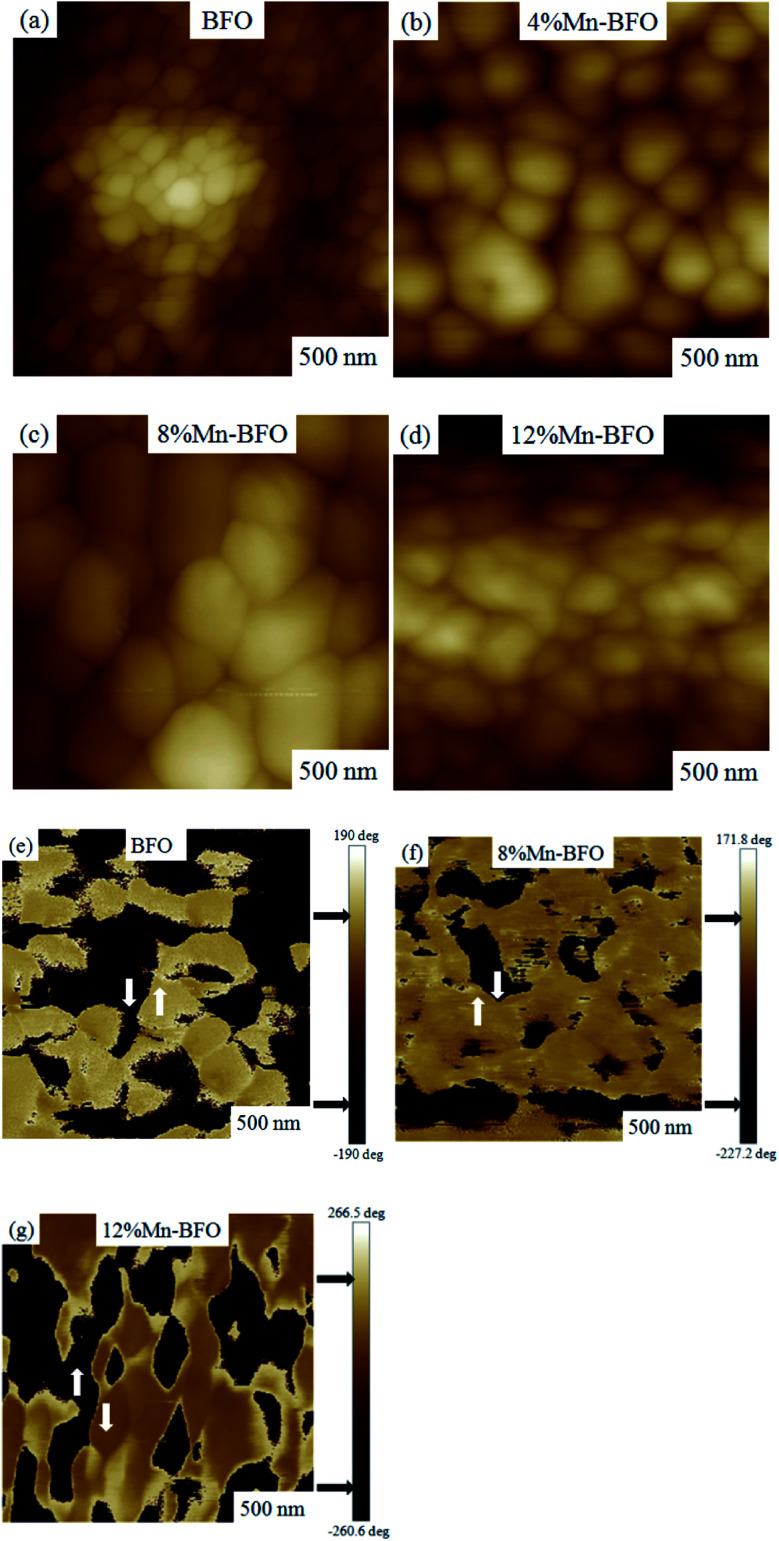
(a)–(d) Surface morphologies of 0–12% Mn doped BFO thin films measured by AFM. (e)–(g) PFM phase images of BFMO thin films.

Fig. 2(a)–(d) show the surface topographic images of Mn doped BFO thin films test by AFM. It shows that the undoped BFO film is uneven and has a little undulating surface, which could impair the properties of the films severely. By contrast, the BFMO thin films exhibit very dense, pore free and uniform microstructure. Thus the substitution of Mn is found to be beneficial to enhance the microstructure of the films. The surface of Mn-doped BFO films were the root-mean-square (RMS) surface roughness values observed for Mn-doped BFO films (with *x* = 0.00, 0.04, 0.08 and 0.12) to be 15.6, 11.2, 8.6 and 10.4 nm respectively. The values of average grain size obtained from the AFM study are about 34.5, 37.7, 46.8 and 35.6 nm for BiFe_1−*x*_Mn_*x*_O_3_ (*x* = 0, 4%, 8% and 12%) thin films. The grain size of the BFO film trend with Mn-doping, which was in agreement with the XRD results (Table 2). The grains of BFMO thin films was found to be increased with increase in Mn doping content (*x* = 0, 0.04, 0.08). A similar increase in grain size on Mn substitution was reported by Kim *et al.* and Choi *et al.*^19–21^ Obviously, a sudden decrease in the grain size was observed in BiFe_0.88_Mn_0.12_O_3_ thin film. This is because in 12% Mn doped BFO thin films, excessive addition of Mn may cause densification problems, thus reducing grain growth.

**Fig. 3:**
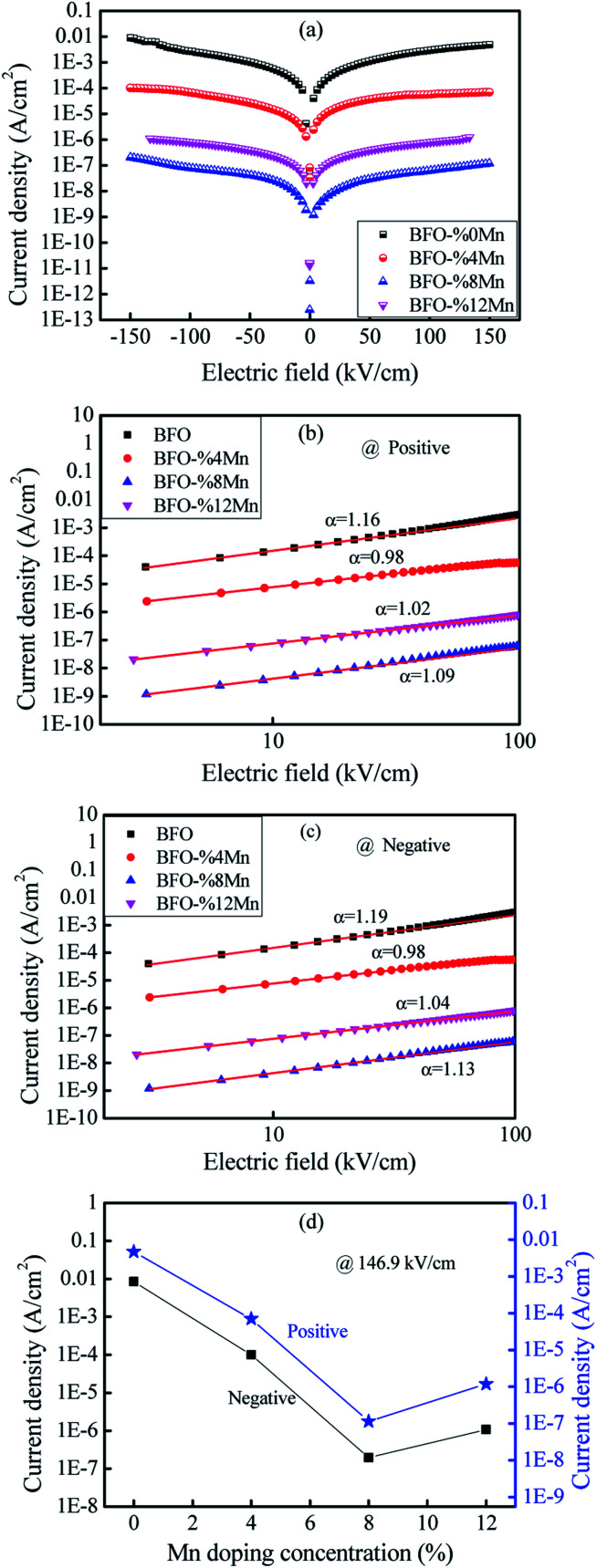
(a) Current density *versus* electric field characteristic of BFMO thin films using logarithmic plots. (b) and (c) log(*J*) *vs.* log(*E*) plots of Mn substituted BFO thin films. (d) The trend of leakage current density with Mn doping concentration.

**Fig. 4:**
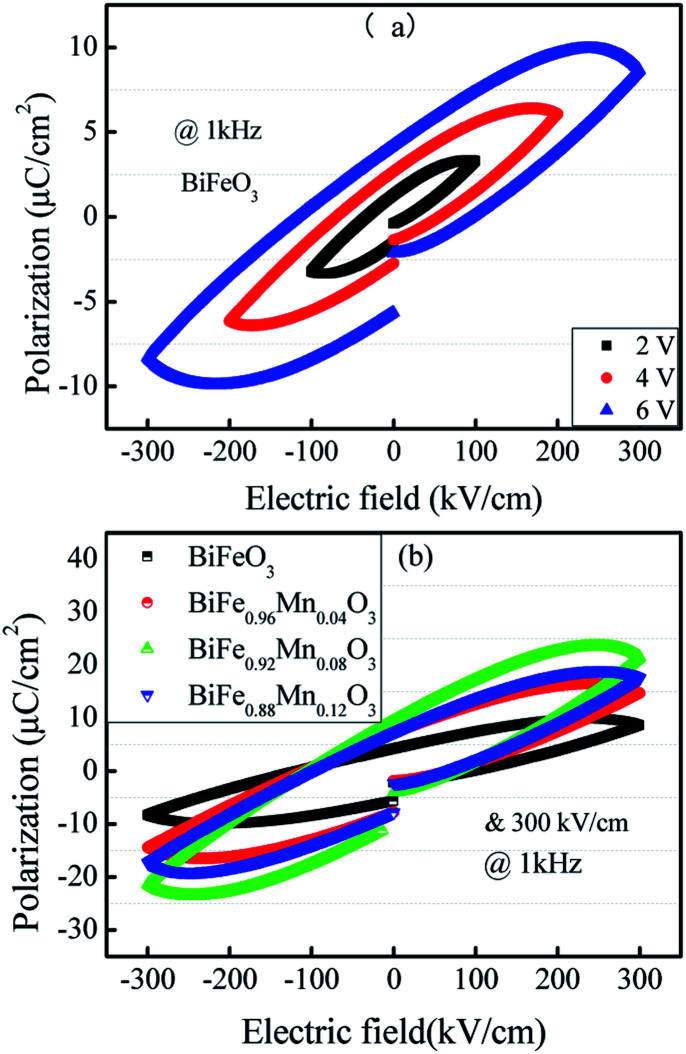
Ferroelectric polarization characterizations (a) and (b) of the Mn-doped BFO films.

**Fig. 5:**
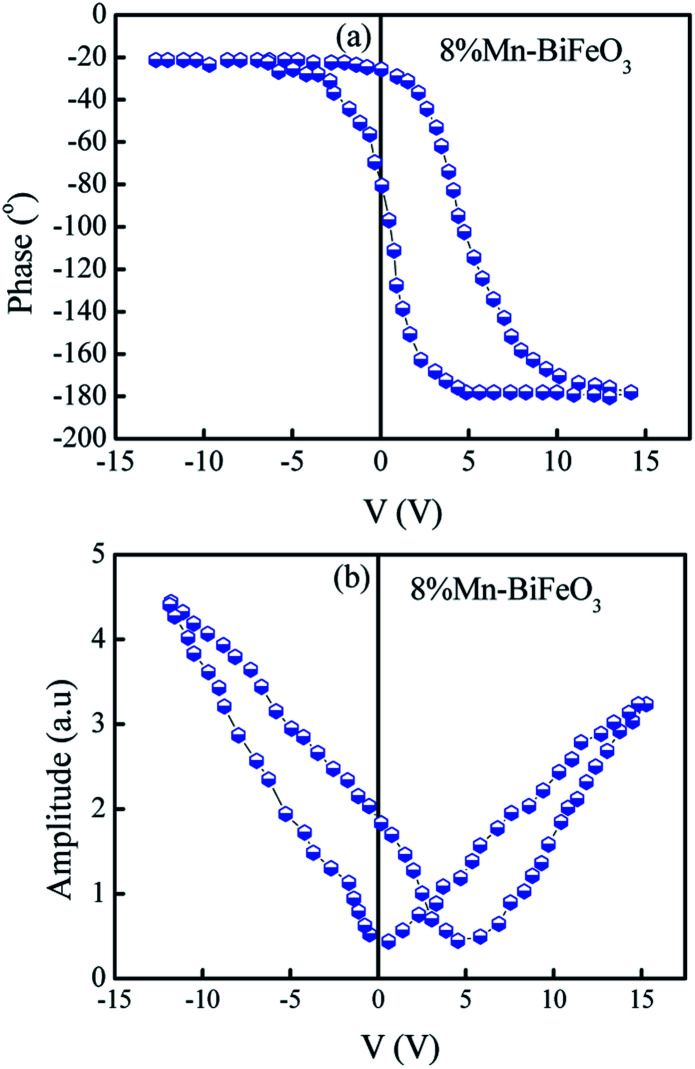
Piezoresponse phase (a) and piezoresponse amplitude (b) *versus* applied voltage loops, measured with an AC driving voltage *V*_ac_ = 5 V.

The out-of-plane PFM phase images of the BFO, 8% Mn–BFO and 12% Mn–BFO films are shown in Fig. 2(e)–(g), respectively. The ferroelectric domain structures of the BFO and 12% Mn–BFO exhibit a fractal growth habit, a domain size similar to the grain size and upward or downward polarization within each domain (Fig. 2(e)). The domain structures of the 8% Mn–BFO show more homogeneous domains and the domains are pinned at the ground boundaries (Fig. 2(f)). The density of the domain walls in the 8% Mn–BFO is less than in the BFO and 12% Mn–BFO. Therefore, it is expected that there is a lower leakage of current and large residual polarization in the 8% Mn–BFO (Fig. 4 and 5) since certain domain walls in the BFO and 12% Mn–BFO are much more conductive than the domains themselves.^22^ In addition, there exist three types of domain walls, namely those that separate domains 71°, 109° and 180° different in polarization.^23^ Domain patterns can develop with either a (100)-type plane for 109° walls or a (101)-type plane for 71° walls, respectively.^24^ As shown from the XRD spectra, the polycrystalline films should be a mixture of all three types of domain wall.^25^ The domain size in the BiFe_0.92_Mn_0.08_O_3_ film is larger than that in the BiFeO_3_ film, which could originate from the tensile strain enhanced by the Mn doping, the bigger grain size or even the misfit dislocation between the film and the substrate.^26^ The domain structure and the density of the domain walls of the BFMO also suggests that it is easier to polarize than the BFO.

### 3.3. Leakage current analysis

Fig. 3(a) presents the typical leakage current behavior (log *J*–*E* curves) of BFMO films. The measured leakage current density of BFMO (*x* = 0, 0.04, 0.08, 0.12) thin films is 9.04 × 10^−2^ A cm^−2^, 1.02 × 10^−4^ A cm^−2^, 1.14 × 10^−7^ A cm^−2^ and 1.10 × 10^−6^ A cm^−2^ at an applied electric field of 146.9 kV cm^−1^, repectively. The doped BFMO (*x* = 0.04, 0.08) thin films exhibit lower leakage current density compared to the undoped BFO thin film. Obviously, as the applied electrical field increases, the leakage current increases. It is worth noting that with the increase of Mn content (0–8%), the leakage current density curves gradually decrease. The two following factors can explain this phenomenon: (i) with the increase of Mn doping, the conversion of Fe^2+^ to Fe^3+^ ions may be promoted. Generally speaking, the less Fe^2+^ ions, the smaller the oxygen vacancy concentration corresponding to the film, which is similar to the results reported by Yoo *et al.*^27^ With the addition of Mn into BFO cells, the valence states of Mn, Fe and O ions react in BFO-based thin films. Oxygen vacancies act as “transition bridges” to achieve charge balance in BFO-based thin films and the reaction equation between them is as follows:^28^2
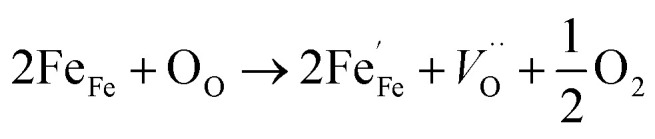

3 Mn^3+^ + Fe^2+^ → Mn^2+^ + Fe^3+^

It can be seen from the formula that Fe^3+^ becomes more stable after Mn doping and limit the movement of oxygen vacancies.^29^ (ii) The increase of (Mn_Fe^3+^_^3+^)* can suppress Fe^3+^ turning into Fe^2+^ by doping of trivalent ions (Mn^3+^) in BFO films.^30^ More significantly, the leakage current density does not present a monotonous decreasing trend with the increase of Mn doping content. As Mn doping content increased from 8% to 12%, the leakage current density began to become larger for 1.10 × 10^−6^ A cm^−2^. This is because the increase in Mn content causes a gradual transformation of BFMO thin films from an insulator to semiconductor and also increase its density of free electrons in same fashion.^31^

Noticing that, the BiFe_0.92_Mn_0.08_O_3_ thin film showed the lowest current density in the low electric field region (*E* < 150 kV cm^−1^) as shown in the Fig. 3(d). Based on the AFM results, a flat and dense surface should be a source of lower leakage current in low electric field. From internal factors, a flat and a dense surface indicate that the grain size is more uniform, so the defect can be reduced. From extrinsic factors, the flat thin films surface could result to a better Pt/BFMO interface leading to the decrement of the leakage current.^29^ We also found that in the low electric field area, the leakage current of Mn-doped BFO films are lower than the undoped one.

To further study the source of leakage current, we analyzed the leakage mechanism of BFMO films. Fig. 3(b) shows the *J*–*E* plots of BFMO thin films in logarithmic scales during the positive process. The plots reveals a near linearity in the range of applied electric fields and they obey the power law of *J* ∝ *E*^*α*^. We can judge the leakage mechanism of the samples by the slope of *α*.^32,33^ Ohmic conduction and space charge limited conduction (SCLC) are the two most common leakage mechanism with the fitting slope of 1 and 2, respectively.^34^ The slope under the low electric field is 1.22, 0.98, 1.03, 1.19 for the samples with *x* = 0%, 4%, 8%, 12% respectively. Which indicates that the ohmic conduction is dominant conduction mechanism in these samples. Similar analyses were conducted to determine the dominant leakage mechanism in the samples during the negative. The fitting results for BFMO (*x* = 0, 0.04, 0.08, 0.12) thin films are show in Fig. 3(c), in which a linear fitting with slope ≈ 1 would suggest the ohmic mechanism during the negative. It is shown that the free carriers in BFMO films play an important role in this stage, and there is no space charge effect.^35^ Therefore, at low electric field, the decrease of leakage current is not due to the decrease of BFO defect number, but mainly due to the inhibition the transformation of Fe^3+^ into Fe^2+^ by Mn doping, and the interface effect plays a major role.

### 3.4. Hysteresis analysis

Fig. 4(a) shows the hysteresis loops at different applied voltages for same frequency. It can be seen that the hysteresis loop is strongly dependent on the applied voltage. For the applied voltage lower than 6 V (∼300 kV cm^−1^), although a hysteresis loop could be observed, the polarization was not saturated around the maximum of the applied voltage, indicating that polarization switching is incomplete. As the applied voltage increased, the reversal proceeded more completely. At 6 V (∼300 kV cm^−1^), the relatively saturated loop was observed.

Fig. 4(b) shows the *P*–*E* hysteresis curves of Mn-doped BFO films with different Mn concentrations at the same electric field. The ferroelectric polarization and other parameters have been summarized in Table 3. It is evident that ferroelectricity of BFMO thin films has been greatly enhanced with the increase in Mn substitution from 0% to 8%, whereas the *P*_r_ of 12% Mn substituted BFO thin films is smaller. The lower residual polarization value and non saturated behavior of 12% Mn doped BFO thin films is associated with the existence of large leakage current component.^21^ It also reported by Das and his co-workers that high leakage characteristics of BFO ceramics could produce difficulty in attaining saturated polarization.^36^

**Table 3:** Different parameters calculated from ferroelectric and optical properties of BFMO thin films

Samples	2*P*_s_ (μC cm^−2^)	2*P*_r_ (μC cm^−2^)	2*E*_c_ (kV cm^−1^)	Direct band gap (eV)	Indirect band gap (eV)
BFO	19.86	9.8	191.54	2.12	2.26
BF_0.96_M_0.04_O	33.02	15.5	151.96	2.0	2.22
BF_0.92_M_0.08_O	47.12	19	134.76	1.92	2.20
BF_0.88_M_0.12_O	37.84	14.6	196	1.97	2.23

The remnant polarization (2*P*_r_) and coercive field (*E*_c_) increase with the elevation of Mn content from 0% to 8% in BFMO thin films. This increase in 2*P*_r_ value with the increase in Mn content from 0 to 8% can be attributed to combine defect of (i) lowering of defects and (ii) increase in the grain size. Space charges such as oxygen vacancies 
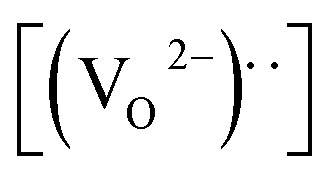
 and acceptors [(A_Fe^3+^_^2+^)*] such as [(Fe_Fe^3+^_^2+^)*] form a defect polarization which can align along the direction of spontaneous polarization alleviates the domain pinning.^37,38^ The substitution of Mn at B site in BFO matrix suppresses the content of defect complexes of 
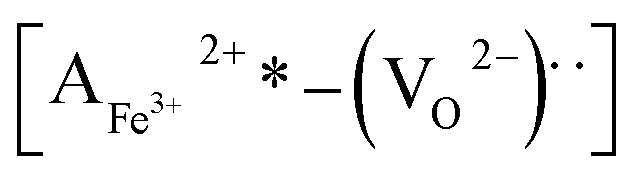
 by suppressing oxygen vacancy concentration, which in turn resulted in higher 2*P*_r_ value.^39^ It is well known that grain size dependent domain structure, domain nucleation and domain mobility greatly influence the ferroelectric properties of ferroelectric thin films.^40^ The grain boundaries act as a pinning center for polarization and produce hindrance of polarization switching. Thus, the small grain size BFMO thin films experience more suppression of ferroelectric character whereas polarization switching is much easier inside the larger grain. The increase in the tetragonality of a crystal structure with increase in Mn doping could be another reason for the enhancement of ferroelectricity as propounded by Ederer *et al.*^41^ This possibility can be easily ruled out as in our experiment, no other phases appeared, as shown in Fig. 1 XRD. Table 3 summarizes the different parameters obtained from the (*P*–*E*) loops of BFMO thin films. The sudden decrease in the remnent polarization 2*P*_r_ value of 12% Mn doped BFO thin film is attributed to decrease in the grain size. A small conveyance in polarization hysteresis loops along the direction of the positive field in BFMO thin films can be imputed of actors like crystallographic defects, work function difference and thermal history between top and bottom electrodes.^42,43^

The piezoelectric response of the 8% Mn–BFO thin film has been studied simply by piezoresponse force microscopy. Typical butterfly loops were observed for 8% Mn–BFO sample, as presented in Fig. 5(a) and (b). Local piezoelectric phase hysteresis loops and amplitude–voltage loops were also recorded at fixed location as a function of ±15 dc bias superimposed on ac modulating voltage. Complete phase reversal of about 180° and piezo-actuation amplitude variation of more than 4.5 mV under the dc bias of ±5 V reflects the better ferroelectric properties of 8% Mn–BFO thin films important for practical applications of interest.^44^

### 3.5. Band-gap analysis

**Fig. 6:**
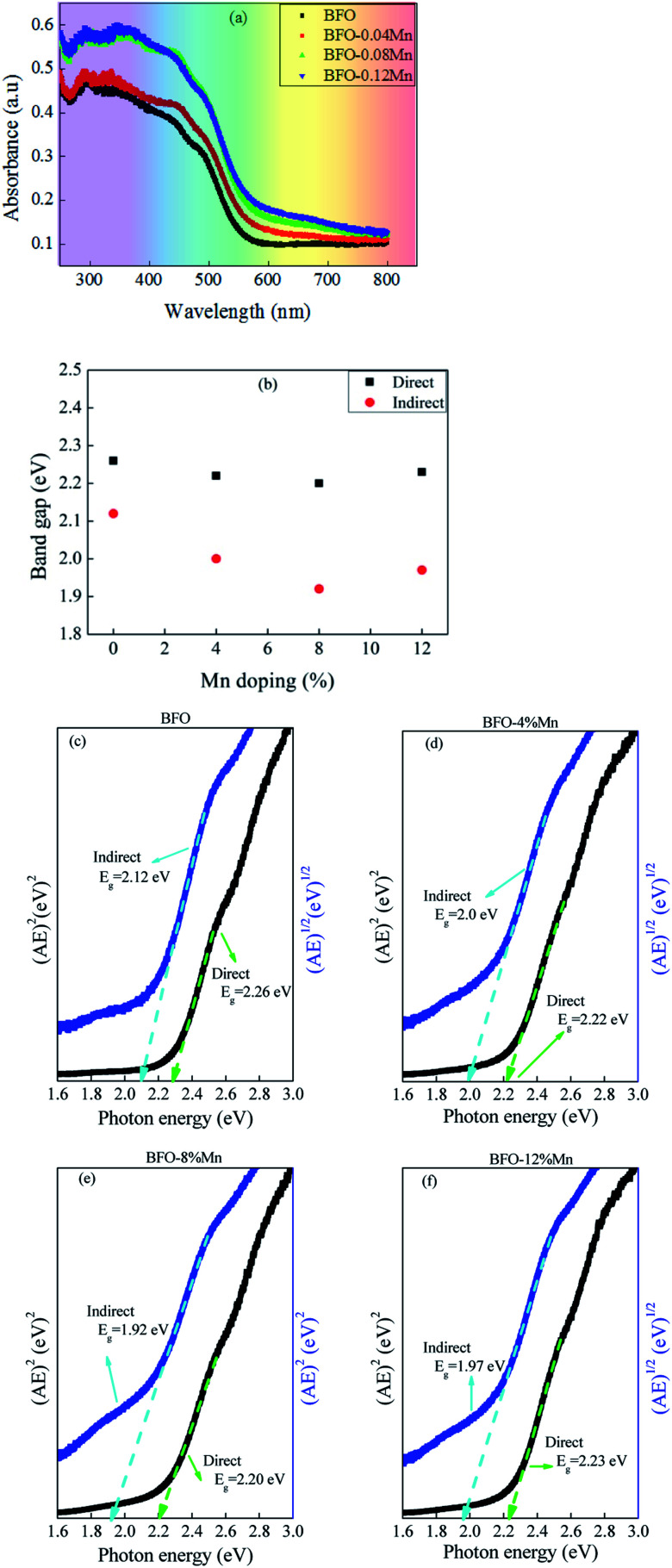
Optical properties of Mn-substituted BFO. (a) Absorption spectrum of Mn-substituted BFO films determined by integral sphere spectrophotometer. (b) Calculated direct and indirect band gap of BFMO samples. (c to f) (*αhν*)^2^ and (*αhν*)^1/2^ Tauc plots of Mn-substituted BFO films used for determining the direct and indirect band gap.

The most direct and perhaps the simplest method for probing the band structure of semiconductors is to measure the absorption spectrum. Fundamental absorption, which manifests itself by a rapid rise in absorption, can be used to determine the energy gap of the semiconductor. To investigate the influence of Mn doping on the optical absorption of BFO, the absorption optical spectra of pure BFO and Mn-doped BFO samples were measured at room temperature, as shown in Fig. 6(a). The absorption band edge of BFO thin film appeared at 572 nm, which was similar to those previously reported,^45,46^ indicating that BFO could respond to visible light for photocatalytic reaction. The fundamental absorption edge is seen to be shifted towards higher wavelength with increasing Mn-doping concentration of the BFMO thin films (Fig. 6(a)). Compared to pure BFO, the Mn-doped BFO samples exhibited enhanced absorption capability especially in the visible light region, and the absorption intensity became gradually stronger as increasing the Mn dopant content. Moreover, the band gap could be calculated from the plot of the Kubelka–Munk function:^47^

4 (*αhν*)^*n*^ = *A*(*hν* − *E*_g_)

where *α* is the absorption coefficient given by Ab/*t*, where Ab is absorbance and *t* is thickness of the film, *h* is Planck constant (*h* = 4.14 × 10^−15^ eV s), *ν* is photon frequency, *A* is a constant, *E*_g_ is band gap energy, and *n*, a number equal to 2 for direct band gap semiconductors and 1/2 indirect band gap semiconductors.^48^ Here we use absorbance to calculate the band gap. Whether Ab or *α* is used, the coefficient *A* is different and has no effect on *E*_g_. Fig. 6(c–f) shows the Tauc plots in the form of (*αhν*)^*n*^*versus* photon energy for direct and indirect band gap semiconductors. By extrapolating the linear part of the absorption curve to the abscissa, the band gap can be determined according to the particular model used.^49^ The slope of the linear part suggests the estimated values of direct and indirect band gap of Mn:BFO films as 2.26 eV, 2.22 eV, 2.2 eV, 2.23 eV and 2.12 eV, 2.0 eV, 1.92 eV, 1.97 eV for the pure BFO, 4% Mn–BFO, 8% Mn–BFO and 12% Mn–BFO samples, respectively. With increasing Mn substitution, this transition branch becomes smeared and better described by an indirect allowed transition model, which is similar to the results of La doped BFO reported by Lu You *et al.*^49^ It should be noted that at room temperature, the phonon absorption branch usually dominates the (*αhν*)^1/2^ plot.^50^ For pure BFO, the linear portions of the direct and indirect Tauc plots almost overlap with each other, implying that this electronic transition contains both direct and indirect features, which is consistent with the flatness of the valence band edge of BFO.^51^ Mn substitution results in effective separation of the direct and indirect portions around this transition, which is most pronounced at 8% concentration. It is observed that while the direct transition remains almost unchanged, the indirect transition strongly red-shifts with increasing Mn concentration (Fig. 6(b)). All these observations point to the fact that Mn substitution makes the band–edge transitions more indirect, which directs us to look into the photocarrier dynamics in these films. With the increase of the Mn dopant content, the band gap of doped samples was gradually decreased, and the band gap energy is minimum for the 8% Mn-doped BFO thin film, in turn leading to a higher absorption capability. Undoubtedly, the enhanced absorption property of Mn-doped BFO would probably improve the photocatalytic activity of BFO, as discussed below. The smaller band gap values of the films predict a possibility of higher absorption of visible light that may lead to potential photocatalytic application in photovoltaic devices. Combined with the change of residual polarization value of doped bismuth ferrite films (Table 3), it is found that the optical band gap decreases with the increase of residual polarization value.

## 4. Conclusions

In summary pure and Mn doped BFO thin films were successfully prepared on Pt/Ti/SiO_2_/Si and ITO/glass substrates by the solution-gelation technique. Structural characterization by X-ray diffraction revealed that all samples exhibited a rhombohedral structure with (111) preferred orientation and without impurity phase. It is observed that improved ferroelectric property and leakage current density in 8% Mn-doped BFO thin film due to a decrease in the oxygen vacancy density, a stabilization of the perovskite structure, and increase in the grain size. The absorption spectrum for BFMO (*x* = 0, 0.04, 0.08) thin films presents a significant red shift and moves towards the visible region. Thus, photovoltaic behaviors are attributed to the narrower band-gap. This work gives insight into the relationship between ferroelectric remnant polarization and band-gap and found that the optical band gap decreases with the increase of residual polarization. This also provides an available way to exploring the mechanism of ferroelectric photovoltaic, getting more extensively applied in the new photovoltaic cells and other novel photoelectronic devices.

## Conflicts of interest

There are no conflicts to declare.

## Supplementary Material
